# Genome‐wide investigation of a neuropathological cohort offers novel insights in the genetic risk underlying hallmark and co‐morbid lesions implicated in Alzheimer’s disease

**DOI:** 10.1002/alz.092254

**Published:** 2025-01-03

**Authors:** Celeste Laureyssen, Klara Gawor, Jasper Van Dongen, Fahri Küçükali, Marta J Koper, Alicja Ronisz, Markus Otto, Christine von Arnim, Philip Van Damme, Rik Vandenberghe, Dietmar Rudolf Thal, Kristel Sleegers

**Affiliations:** ^1^ Department of Biomedical Sciences, University of Antwerp, Antwerp Belgium; ^2^ Complex Genetics of Alzheimer’s Disease Group, VIB Center for Molecular Neurology, VIB, Antwerp Belgium; ^3^ Laboratory for Neuropathology, KU Leuven, Leuven Belgium; ^4^ Complex Genetics of Alzheimer’s Disease group, VIB‐UAntwerp Center for Molecular Neurology, Antwerp Belgium; ^5^ University of Antwerp, Antwerp Belgium; ^6^ Department of Neurology, Ulm University Hospital, Ulm Germany; ^7^ University Medical Center Göttingen, Göttingen Germany; ^8^ Department of Neurosciences, KU Leuven, Leuven Belgium; ^9^ UZ Leuven, Leuven Belgium; ^10^ KU Leuven, Leuven Belgium; ^11^ Alzheimer Research Centre KU Leuven, Leuven Brain Institute, Leuven Belgium

## Abstract

**Background:**

Alzheimer’s disease (AD) is a heterogenous disease with a strong heritability. Genetic studies are of irreplaceable value in elucidating the mechanisms that underly this disease. The classical genome‐wide association studies (GWAS) rely on ever‐increasing sample sizes and utilize clinical AD diagnosis to investigate genetic risk. Here, we aim to eliminate phenotypic heterogeneity by performing GWAS on a deeply phenotyped neuropathological cohort.

**Method:**

Phenotypic data regarding hallmark and co‐morbid AD lesions was collected on 721 individuals. DNA was extracted from frozen or paraffin‐fixed cerebellar tissue. Subsequently, genetic data was generated using low‐coverage whole‐genome sequencing (lcWGS) followed by imputation based on 1000 Genomes Project reference panel using GLIMPSE. Linear or logistic regression was performed in PLINK correcting for age, gender and the first 3 PCs for every phenotype. Investigated phenotypes include but are not limited to: Amyloid phases, Braak‐NFT stage, cerebral amyloid angiopathy (CAA) severity and type, pTDP presence in CA1 and dentate gyrus, alpha‐synuclein spread/presence, age‐related tau‐astrogliopathy (ARTAG) and Hirano bodies.

**Result:**

We discovered genome‐wide significant hits for well‐established AD lesions such as tau tangles, amyloid plaques and CAA. Furthermore, significant hits for co‐morbid lesions like alpha‐synuclein stages, granulovacuolar degeneration and Hirano bodies (extracellular actin aggregates in the CA1) were found. Suggestive associations were discovered for pTDP and ARTAG. Associated loci included proof‐of‐concept findings such as strong relations between amyloid and CAA with APOE SNPs, but also included several loci that have not been previously linked to the specific phenotype or AD in general.

**Conclusion:**

Implementing phenotypic information on neuropathological lesions in a hypothesis‐free genome‐wide investigation as a preliminary screening rendered genome‐wide significant and suggestive loci which can contribute to a deeper understanding of the individual lesions observed in AD. From our preliminary results we observe the benefits of accurate phenotyping on GWAS power. Despite small sample size, we managed to detect genome‐wide significant associations for AD‐related lesions by eliminating phenotypic heterogeneity. Associated loci will be subjected to gene prioritization and functional validation analysis to determine the causal risk genes. For more commonly investigated hallmark lesions, replication analysis will be performed in publicly available cohorts.

